# Mitophagy and Oxidative Stress: The Role of Aging

**DOI:** 10.3390/antiox10050794

**Published:** 2021-05-17

**Authors:** Anna De Gaetano, Lara Gibellini, Giada Zanini, Milena Nasi, Andrea Cossarizza, Marcello Pinti

**Affiliations:** 1 Department of Life Sciences, University of Modena and Reggio Emilia, 41125 Modena, Italy; anna.degaetano@unimore.it (A.D.G.); giada.zanini@unimore.it (G.Z.); 2 Department of Medical and Surgical Sciences of Children and Adults, University of Modena and Reggio Emilia, 41125 Modena, Italy; lara.gibellini@unimore.it (L.G.); andrea.cossarizza@unimore.it (A.C.); 3 Department of Surgery, Medicine, Dentistry and Morphological Sciences, University of Modena and Reggio Emilia, 41125 Modena, Italy; milena.nasi@unimore.it

**Keywords:** mitophagy, aging, Reactive Oxygen Species, PINK1, mitochondria, Alzheimer, Parkinson

## Abstract

Mitochondrial dysfunction is a hallmark of aging. Dysfunctional mitochondria are recognized and degraded by a selective type of macroautophagy, named mitophagy. One of the main factors contributing to aging is oxidative stress, and one of the early responses to excessive reactive oxygen species (ROS) production is the induction of mitophagy to remove damaged mitochondria. However, mitochondrial damage caused at least in part by chronic oxidative stress can accumulate, and autophagic and mitophagic pathways can become overwhelmed. The imbalance of the delicate equilibrium among mitophagy, ROS production and mitochondrial damage can start, drive, or accelerate the aging process, either in physiological aging, or in pathological age-related conditions, such as Alzheimer’s and Parkinson’s diseases. It remains to be determined which is the prime mover of this imbalance, i.e., whether it is the mitochondrial damage caused by ROS that initiates the dysregulation of mitophagy, thus activating a vicious circle that leads to the reduced ability to remove damaged mitochondria, or an alteration in the regulation of mitophagy leading to the excessive production of ROS by damaged mitochondria.

## 1. Introduction

Mitochondrial (mt) dysfunction is considered a hallmark of aging [1]. Dysfunctional mitochondria are recognized and degraded either by non-selective autophagy or by a selective type of macroautophagy, named mitophagy [2]. This catabolic process allows the degradation of dysfunctional and damaged mitochondria [3,4], with the aim of recycling mitochondrial contents and macromolecules, such as amino acids and preserving ATP production [3,5,6,7,8]. Mitophagy is evolutionarily conserved and has been observed from yeast to mammals. Mitophagy process starts when dysfunctional mitochondria are targeted with specific receptors or adaptors and are engulfed in a double-membrane vacuole named autophagosome. Then, this vesicle fuses with a lysosome, forming an autolysosome in which specific enzymes degrade the organelle. Based on the ability of receptor to recruit ubiquitin, the mitophagy regulatory pathways could be classified as ubiquitin-dependent or ubiquitin-independent (receptor-dependent) [3,9]. This process is triggered by multiple stimuli and can be activated on the basis of cell requirement. Depending on the physiological condition of the cell, mitophagy can be classified in steady-state or basal mitophagy, programmed mitophagy, and stress-induced mitophagy [5,10]. The functions of basal mitophagy are not well understood and the process level differs between cells and tissues. However, it is likely that, in physiological conditions, mitophagy is required for mitochondrial turnover, cellular homeostasis, and metabolic demand [3,10,11,12,13]. Programmed mitophagy is necessary for development and differentiation processes, such as maturation of erythrocytes and cardiomyocytes and for allophagy [5,10,14,15,16]. Finally, stress-induced mitophagy is activated by stimuli, such as oxidative stress, starvation, hypoxia, and loss of the mitochondrial membrane potential (MMP), with the aim of reducing mitochondrial amount and, in turn, decreasing the production of reactive oxygen species (ROS) and oxygen consumption by damaged mitochondria [17].

Several lines of evidence indicate a close correlation between the increase in ROS observed with age and the modulation of age-dependent mitophagy. On the one hand, the study of mitophagy on different model species, from *S. cerevisiae* to *C. elegans*, clearly indicates that the aging process is related to an impairment of the regulation of mitophagy, and that targeting of genes that regulate mitophagy can modulate lifespan. On the other hand, several studies on cellular and in vivo models have shown a close correlation between ROS production, mitochondrial stress, and activation of mitophagy. Since oxidative stress is one of the main drivers of aging, and many diseases characterized by premature aging are characterized by an excess of ROS production or a defect in the scavenging processes of free radicals, it is likely that age-related increase of ROS can play a role in the impairment of mitophagy observed with aging.

In this review, we discuss the main findings linking mitophagy, oxidative stress, and aging, both in physiological aging, and in age-related diseases such as Alzheimer’s disease (AD) and Parkinson’s disease (PD).

## 2. Mitophagy and Its Regulation

**Figure 1:**
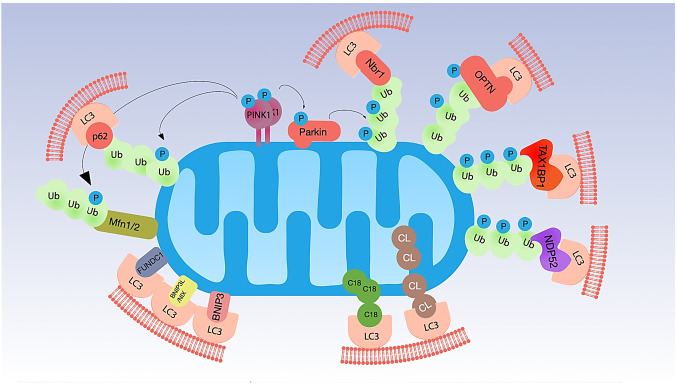
Main molecular mechanisms of mitophagy. Mitophagy is regulated by the interaction of mitochondrial proteins with LC3 through different mechanisms. In the PINK1/Parkin pathway, decreased MMP leads to the accumulation of PINK1 to the OMM. PINK1 phosphorylates both ubiquitin and Parkin. Activated Parkin polyubiquitinates specific proteins on the OMM, making available ubiquitins for PINK1 phosphorylation. The ubiquitinated proteins on the OMM allow the interaction of mitochondria with LC3 through specific adaptors, such as p62, Nbr1, OPTN, TAX1BP1, and NDP52. PINK1 can also phosphorylate Mfn2 and promote its ubiquitination by Parkin and rapid degradation, to prevent fusion of damaged mitochondria with healthy organelles. Besides PINK1/Parkin pathway, mitophagy is triggered by the mitochondrial receptors BNIP3, BNIP3L/NIX, FUNDC1, which can bind directly to LC3. Finally, mitochondrial lipids cardiolipin (CL) and C18-ceramide (C18) can move from the mitochondrial cristae to the OMM, where they interact with LC3.

Mitophagy is an extremely complex and finely regulated process; the detailed description of the molecular mechanisms underpinning mitophagy goes far beyond the purpose of this review For this reason, we will summarize only the pathways that are—or could be—relevant for the aging process (Figure 1).

### 2.1. Ubiquitin-Dependent Mitophagy

The phosphatase and tensin homologue (PTEN)-induced putative kinase 1 (PINK1)–Parkin pathway represents one of the most studied ubiquitin-dependent mechanism of mitophagy. In functional mitochondria, the serine/threonine kinase PINK1 is continually imported to mitochondria through the translocases of the outer and the inner membrane (TOM and TIM complexes) taking advantage of the mitochondrial targeting sequence (MTS). During the import, PINK1 is clipped first by matrix processing peptidases (MPP) and subsequently by a protease of the mitochondrial inner membrane (IMM), the presenilin-associated rhomboid like (PARL). Cleaved PINK1 moves to the cytosol, where is rapidly degraded [2,18,19]. In damaged or aged mitochondria, the decrease of the MMP locks the import of PINK1 in the mitochondrial matrix and its degradation, stabilizing it on the mitochondrial outer membrane (OMM) in a complex with the translocase TOM [2,7,19]. Stabilized PINK1 is auto-phosphorylated and: (i) phosphorylates ubiquitin and poly-ubiquitin, connected in a basal manner to proteins on the OMM, at Ser65, (ii) recruits and phosphorylates the cytosolic E3-ubiquitin ligase Parkin at Ser65. Phospho-ubiquitin, in turn, can recruit Parkin [3,10,20,21,22,23,24]. The active conformation of Parkin polyubiquitinates specific proteins on the OMM, making available ubiquitins for PINK1 phosphorylation, and triggering a feedback loop which leads to the recruitment of other molecules of Parkin on the mitochondrial surface finally activating mitophagy in a feedforward mechanism [24]. The ubiquitinated proteins of the OMM recruit five LC3 interacting region (LIR)-containing autophagy adapters: sequestosome-1 (p62/SQSTM1), Optineurin (OPTN), neighbor of BRCA1 gene 1 (NBR1), nuclear domain 10 protein 52 (NDP52), and TAX1 binding protein 1 (TAX1BP1). Through the LIR region, these ubiquitin-binding receptors recognize and bind LC3 driving mitochondria to mitophagy [2,25]. The PINK1/Parkin pathway is strictly interconnected with molecules and pathway regulating mitochondrial dynamics, such as Mitofusin (MFN) 1 and 2, GTPase of OMM involved in mitochondrial fusion MFN1 and MFN2 are highly susceptible to Parkin ubiquitination. MFN2 mediates the recruitment of Parkin to damaged mitochondria; PINK1 phosphorylates Mfn2 and promotes its ubiquitination by Parkin [26]. Thus, PINK1/Parkin activation causes their rapid ubiquitination and degradation, which prevents fusion of damaged mitochondria with healthy organelles [26].

In addition to Parkin, several other ubiquitin E3 ligases are able to ubiquitinate proteins on the mitochondrial surface: SMAD ubiquitination regulatory factor 1 (SMURF1), Glycoprotein 78 (Gp78), mitochondrial E3 ubiquitin protein ligase 1 (MUL1), HECT, UBA, and WWE domain-containing protein 1 (HUWE1), E3 ubiquitin-protein ligase SIAH1 (SIAH1), and Ariadne RBR E3 ubiquitin ligase homolog 1 (ARIH1) [5,9,10,27,28,29,30,31,32,33,34,35,36]. The ubiquitin chains generated recruit autophagy adaptors, such as p62, NDP52, and OPTN. Finally, other molecules such as unc-51-like autophagy activating kinase 1 (ULK1) mediates the biogenesis of the phagophore [9,25].

### 2.2. Ubiquitin-Independent Mitophagy (Receptor-Dependent)

An alternative mechanism to ubiquitin-dependent mitophagy takes advantage of protein receptors encompassing LIR motif and able to interact directly with LC3 or other autophagy-related proteins (ATGs) such as GABARAP in a ubiquitin-independent way.

In OMM, FUN14 domain containing 1 (FUNDC1), BCL2 interacting protein 3 (BNIP3), and NIP3-like protein X (NIX, also known as BNIP3-like, NIX/BNIP3L) are involved in the induction of mitophagy under stress condition, such as hypoxia [37,38,39]. The interaction of this group of protein with LC3/GABARAP is generally mediated by their phosphorylated/dephosphorylated status.

FUNDC1 contributes to mitophagy during hypoxia in mammalian cells by directly binding to LC3 [2,27]. In normoxic conditions, FUNDC1 is phosphorylated at Ser13 by casein kinase II (CK2) and at Tyr18 by SRC kinase, and this prevents its interaction with LC3 and, in turn, the activation of mitophagy pathways [27]. In hypoxic conditions, phosphoglycerate mutase 5 (PGAM5) dephosphorylates FUNDC1 at Ser13, while serine/threonine-protein kinase ULK1 phosphorylates it at Ser17. Dephosphorylation of FUNDC1 mediated by PGAM5 improves its interaction with LC3 [28]. FUNDC1 is also involved in the intercross communication between mitochondrial fission and fusion and mitophagy. Specifically, during physiological conditions, FUNDC1 can bind the Dynamin-like 120 kDa protein (OPA1, GTPase involved in fusion and fission processes) to the inner surface of OMM taking advantage of Lys70. Conversely, under stress conditions, this interaction is reduced and FUNDC1 can also recruit Dynamin-1-like protein (DNM1L, GTPase involved in fission) from cytosol [29].

NIX and BNIP3 are proteins with homology to BCL2 in the BH3 domain. NIX (also called BNIP3L) can trigger mitophagy under both physiological and hypoxic conditions. During development, NIX plays an important role in the maturation process of erythrocytes, eliminating mitochondria [16,19]. BNIP3 and NIX mRNA and BNIP3 protein are induced by hypoxic environment in a wide range of human epithelial, endothelial, and macrophage cell lines but not in fibrosarcoma or lymphocyte cell lines [30]. Moreover, NIX and BNIP3 are induced at the transcriptional level also in CHO-K1 cells [31]. It is possible that BNIP3 is a direct target of hypoxia-inducible factor 1-alpha (HIF1α) [30,31]. In addition to its involvement in ubiquitin-independent mitophagy, NIX acts as a substrate of Parkin, recruiting NBR1 and finally triggering mitophagy [32].

Other mitophagy protein receptors of OMM are autophagy and beclin 1 regulator 1 (AMBRA1), FKBP prolyl isomerase 8 (FKBP8), and BCL2 like 13 (BCL2L13).

### 2.3. Mitophagy Triggered by Lipid Receptors

In addition to protein receptors, mitophagy can also be promoted by lipids such as Cardiolipin (CL), C18-ceramide, and SREBF1 [17]. CL triggers mitophagy moving from mitochondrial cristae (where is located in normal conditions) to OMM where interacts with LC3 taking advantages from LIR motif [17,33,34]. Interestingly, CL is particularly prone to peroxidative attack by ROS, and CL peroxidation has been shown to play a critical role in several physiopathological situations [35], including neurodegenerative diseases [36].

## 3. Effects of Oxidative Stress on Mitophagy

Mitochondria are the main source for cellular ROS [37]. NADH: ubiquinone oxidoreductase, or complex I (CI) is the main producer of superoxide anion (O_2_^•−^) in the mitochondrial (mt) matrix [38]. The coenzyme Q:cytochrome c—oxidoreductase or complex III (CIII) is the main source of O_2_^•−^ in the intermembrane space [39]. Mitochondria detoxifies the excess of O_2_^•−^ by means of manganese superoxide dismutase (MnSOD or SOD2), which is located in the mt matrix, and of the copper-zinc SOD (CuZnSOD or SOD1), which is located in the intermembrane space. The product of SOD-mediated reactions is hydrogen peroxide (H_2_O_2_), which is less reactive and can diffuse across mitochondrial membranes. Because of its low reactivity, its relative specificity for cysteine residues, and its capability to diffuse across membranes, H_2_O_2_ can also act as a second messenger [40]. However, uncontrolled levels of H_2_O_2_ lead to hydroxyl radical (OH^•^) formation. For this reason, H_2_O_2_ levels are tightly regulated by robust detoxification systems. In the cytosol, it is converted to water primarily by catalase, while in the mitochondria it is detoxified by peroxiredoxin -3 (Prx3) and -5 (Prx5), by the Glutathione peroxidase 1 (GPx1) and Glutaredoxin 2 (Grx2) [41].

Oxidative stress occurs when the steady-state levels of ROS surpass their catabolism or detoxification, and can regulate mitophagy at multiple levels. Stress conditions stimulating ROS production, such as hypoxia, nutrient starvation, and ischemia/reperfusion (IR) can cause oxidative damage to mitochondria. One of the early responses to excessive ROS is to induce mitophagy, which can reduce oxidative damage and ROS production [42] and multiple lines of evidence have proved that ROS interacts with both ubiquitin- dependent and receptor-dependent mitophagy pathways. Generation of ROS within mitochondria using the mitochondrial-targeted photosensitizer mt KillerRed (mtKR) causes increased ROS levels in the mt matrix, the loss of MMP, and the activation of Parkin-dependent mitophagy. The overexpression of the mt antioxidant proteins, like mtSOD2, abolishes this effect [43]. ROS determines the recruitment of Peroxiredoxin-6 (PRDX6) to damaged mitochondria, where it controls ROS homeostasis in the initial step of PINK1-dependent mitophagy [44]. Although mtROS resulted not required for mitophagy [45] and not involved in mitochondrial translocation of Parkin, O_2_^•−^ has been shown to be drive the progression of Parkin/PINK1-dependent mitophagy, once Parkin has translocated to mitochondria [46]. Mild oxidative stress selectively triggers mitophagy in the absence of autophagy, which is dependent on Dynamin-1-like protein (Drp1) [47].

Changes in redox balance of the cell can also affect mitophagy by modifying mitochondrial dynamics. When reduced glutathione (GSH) is oxidized to oxidized glutathione (GSSG), Mfn forms oligomers via disulfide bond formation and enhances membrane fusion [48]. Conversely, S-nitrosylation of Drp1 determines mitochondrial fragmentation [49]. The effect of oxidative stress and redox imbalance on PINK1/Parkin is less obvious and clear. The oxidation of Parkin has been reported to inhibit [50,51] or stimulate [52,53] the activity of E3-Ub ligase, depending on the model taken into account. It is likely that these opposite effects are due to different cysteine residues modified.

Another emerging link between oxidative stress, mitophagy, and aging is provided by the regulation of the ataxia-telangiectasia mutated (ATM)/Denitrosylase S-nitrosoglutathione reductase (GSNOR) axis. The downregulation of GSNOR during senescence and human aging promotes mitochondrial nitrosative stress, nitrosylation of Drp1 and Parkin, and impairment of mitochondrial dynamics and mitophagy [54]. GSNOR is also induced at the translational level in response to H_2_O_2_ and mt ROS [55].

Methionine is a sulfur-containing amino acid susceptible to reversible oxidation. It has been recently demonstrated that the mitochondrial matrix protein methionine sulfoxide reductase B2 (MsrB2) is a Parkin substrate, needed for mitophagy induction [56]. In condition of high oxidative stress, Parkin is oxidized at Met192, leading to protein inactivation and inhibition of mitophagy. MsrB2 released from damaged mitochondria reduces oxidized Met192, restoring Parkin function. Interestingly, Met192 is mutated in familial, early onset forms of PD [57], a typical age-related disease (see below), and levels of MsrB2 declines with age [58]. Thus, an imbalance in this axis due to the impaired capability to restore reduced Met192 could contribute to the age-related mitophagy dysregulation.

ROS can also modulate mitophagy at transcriptional level [59]. A crucial transcription factor for response to oxidative stress is the nuclear factor (erythroid-derived 2)-like 2 transcription factor (Nrf2). In unstressed conditions, Nrf2 is sequestered in the cytoplasm by the kelch-like ECH-associated protein 1 (Keap1)-Cul3 complex and targeted for ubiquitin-mediated degradation. Under oxidative stress conditions, Keap1 cysteines are oxidized, and Keap1 cannot ubiquitinate Nrf2, which is free to translocate to the nucleus and activate antioxidant response [60]. A tight interplay exists between Nrf2 and mitophagy upon oxidative stress. Nrf2 regulates PINK1 expression under oxidative stress conditions [61], and pharmacological inhibition of Keap1 triggers mitophagy [62]. Furthermore, autophagic degradation of Keap1 mediated by p62 activates Nrf2, which in turn increases the transcription of p62 gene [63,64]. In a model of liver damage, p62 can also promote mitochondrial ubiquitination in Parkin-independent mitophagy, by recruiting Keap1 and another cullin, E3 ubiquitin-protein ligase RBX1 (Rbx1), to mitochondria [65]. In glioblastoma, NRF2 activates NIX in conditions of hypoxia and oxidative stress, and silencing NIX promotes the production of superoxide under hypoxia, likely mediated by dysfunctional mitochondria [66]. Since the FUNDC1 phosphatase PGAM5 is a substrate for Keap1 [67], it is also possible that FUNDC1-dependent mitophagy is regulated by Keap1/Nrf2 axis, even if this hypothesis is still to be proved.

Another crucial transcriptional factor that modulates mitophagy in oxidative stress conditions is HIF-1α [30]. Low O_2_ tension and subsequent increase of ROS inhibit prolyl hydroxylase (PHD) responsible for HIF-1α degradation [68]. High ROS levels can also stabilize Sentrin/SUMO specific proteases (SENPs) that enhance HIF-1α transcriptional activity [69]. Thus, HIF-1α induces the transcription of mitophagy receptors BNIP3 and NIX, producing a metabolic adaptation to a hypoxic environment [30]. Notably, the absence of BNIP3 in mammary cancer causes accumulation of dysfunctional mitochondria and elevated mtROS that upregulates HIF-1α and HIF-1α target genes, including those involved in cancer aggressiveness [70]. This observation further underlines that efficient turnover of mitochondria mediated by mitophagy is crucial in preventing ROS-mediated damage.

Mitophagy is also regulated by the action of Histone acetyltransferases (HATs) and histone deacetylases (HDACs). HATs acetylate conserved Lys on target proteins, while HDAC deacetylates Ac-Lys. Two classes of HAT and three classes of HDAC exist [71]. Of particular interest in the regulation of mitophagy is the class III HDAC, a family of NAD+ dependent deacetylases, known as sirtuins [72]. Seven sirtuins have been identified in humans (SIRT1-SIRT7) with different subcellular distribution; SIRT3, SIRT4, and SIRT5 are located in mitochondria [73]. SIRT 3, the best characterized mitochondrial sirtuin, is involved in the regulation of mitophagy in different ways [74]. By activating LKB1, SIRT 3 promotes the activation of AMPK–mTOR pathway, which in turn leads to autophagy. Furthermore, SIRT3 triggers mitophagy via deacetylation of FOX O3 under oxidative stress conditions. Once deacetylated, FOXO3 translocates to the nucleus, where it promotes the transcription of NIX, Bnip3, and LC3 [75]. Finally, SIRT 3 can mediate an antioxidant response by deacetylating superoxide dismutase 2 (SOD2), a crucial mitochondrial antioxidant enzyme, in two critical residues. A higher activity of SOD2 reduces mtROS, and inhibits Beclin-1. The inhibition of Beclin-1 reduces mitophagy. The activity of sirtuins declines with aging, and this decline can contribute to age-dependent impaired mitophagy [76,77]. The decline of Sirtuin activity could be due to the parallel decline of NAD+ levels with age, as the upregulation of NAD metabolism counteracts age-related diseases, and increasing intracellular NAD^+^ improves mitochondrial quality via mitophagy and reverse cognitive deficits in models of AD [78,79].

Finally, a possible role of ROS in regulating mitophagy is played in immune response, and in particular in natural killer (NK) cells. After viral infection, the majority of effector NK cells undergo apoptosis; ROS triggers BNIP3- and NIX-dependent mitophagy, which in turn promotes the survival of virus-specific NK cells and seeding of memory, by removing dysfunctional mitochondria [80].

## 4. Mitophagy and Oxidative Stress in Physiological Aging

Several lines of evidence strongly suggest that mitophagy plays a role in aging, and recent studies have demonstrated that mitophagy is crucial in delaying physiological aging and age-related disorders, such as neurodegenerative and cardiovascular diseases. Mitophagy decline with aging has been observed in different tissues either from humans or mice, including (but not limited to) myocardium [81] skeletal muscle [82,83] skeletal muscle satellite cells [84], dentate gyrus [85], cultured fibroblasts [86].

A crucial contribution in understanding the crosstalk between aging, ROS, and mitophagy has been given by studies performed in model organisms, such as *Saccharomyces cerevisiae*, *Caenorhabditis elegans,* and *Drosophila melanogaster.* In *S. cerevisiae*, deficiency in non-selective autophagy (atg1Δ) and ubiquitin-independent mitophagy (atg32Δ or atg11Δ) causes accumulation of ROS upon starvation [87,88]. In mitophagy-deficient cells, excessive quantity of mitochondria is not degraded, produce ROS in excess, and spontaneously age [88] suggesting a link between mitophagy, ROS and aging. In *C. elegans*, mitophagy mediated by dct-1, the homolog of BNIP3 and NIX/BNIP3L, plays an important role during aging. DCT-1 is a key mediator of mitophagy and contribute to longevity in stress conditions. Deficiency of dct-1 causes accumulation of mitochondria in young adults, in a way similar to what observed in aged animals. Impairment of mitophagy triggers mitochondrial retrograde signaling, which coordinates the biogenesis and turnover of mitochondria and antagonizes the aging process [89].

The importance of mitophagy in aging of *C. elegans* is also demonstrated by the effects of mitophagy modulation on daf-2 mutants, which are characterized by extended lifespan. Induction of mitophagy in these mutants determines a lifespan shortening. Similarly, altered mitophagy by inactivating dct-1, PINK-1, and pdr-1 (the *C. elegans* Parkin homologs) significantly reduces their lifespan. Although none of these studies has analyzed in depth the consequence of ROS production and oxidative stress, it is interesting to note that dct-1 is transcriptionally regulated by skn-1 and daf-16, the counterparts of mammalian NRF2 and FOXO3, which are crucial regulators of oxidative stress response. In agreement with these observations, it has been reported that the glycoalkaloid tomatidine enhances lifespan in *C. elegans* through ROS-dependent induction of skn-1, which in turn induces mitophagy [90].

Furthermore, mitophagy is activated in reaction to mitochondrial stress in pdr-1, PINK1, and dct-1 dependent manner, as a response to iron starvation upon frataxin depletion. This response is similar to that one to hypoxia, and is involved in the extension of animal lifespan [91]. However, it must be noted that in human cells, loss of iron does not cause depolarization of mitochondria or extensive production of ROS, if compared to electron transport chain inhibitors [92]. Thus, it is possible that it is activated in response to free-iron deficiency stored in the organelle, rather than in response to ROS-induced damage or to metabolic reprogramming induced by HIF-1α.

In *D. melanogaster*, *Pink1,* and *Parkin* mutants are characterized by male sterility, loss of normal mitochondrial morphology and muscle degeneration [93]. In intestinal stem cells, depletion of *Pink1* or *Parkin* alters mitochondrial morphology and density, and results in higher levels of ROS in the intestinal progenitor cells; these changes are associated with an up-regulation of senescence-associated markers [94]. Conversely, the overexpression of *Pink1* and *Parkin* in indirect flight muscles leads to lifespan extension [95].

The importance of excessive ROS production in age-related impairment of mitophagy has been also observed in different mice models. Sarcopenia is one of the most evident phenomena that characterize the aging process, and is strictly associated with mitochondrial dysfunction and oxidative stress. In skeletal muscle, a dramatic impairment of PINK1/Parkin pathway has been observed with aging, and a crucial role of this pathway in counteracting the age-related mitochondrial dysfunction has been demonstrated. Knock out of Parkin in mice leads to an aging-like phenotype of skeletal muscle in adult animals [96], while Parkin overexpression increases mitochondrial enzyme activity, mitochondrial content in skeletal muscle of aged mice, and attenuates age-related oxidative stress [97]. Accordingly, a reduction in Parkin levels has been observed in atrophied muscle of elderly men [98] as well as a reduced expression of PARK2 gene in elderly, inactive women [82]. In the same tissue, an age-dependent decrease of MFN2 has been observed that impacts on mitophagy. Low levels of MFN2 impair mitochondrial fusion/fission regulation and quality control, and favor the accumulation of damaged mitochondria [99]. The subsequent higher ROS production initially activates a feedback loop that promotes mitochondrial turnover through the axis ROS/HIF1a/BNIP3/mitophagy, and minimizes mitochondrial damage. When MFN2 is absent or its levels are too low, mitophagy impairment occurs and age-related mitochondrial dysfunction are exacerbated [99]. The age-related reduction of MFN2 and the impairment of mitophagy impacts also other tissues. MFN2 promotes mitophagy and prevents mitochondrial dysfunction caused by ischemia/reperfusion in murine liver; the age-related reduction of MFN2, along with SIRT1, makes hepatocytes more susceptible to ischemia/reperfusion injury. A similar role has been shown in murine cultured neurons [100]. In chondrocytes, an age dependent reduction of MFN2 causes a reduction in mitochondrial fission, accompanied by dysfunctional mitochondria and oxidative stress [101]. Interestingly, in this model Parkin negatively regulates the levels of MFN2, and an age-related decrease of Parkin causes a post transcriptional increase of MFN2 and hyperfused mitochondria. These observations suggest that a limited production of ROS can activate mitophagy to prevent or reduce mitochondrial damage. When the production of ROS is excessive, activation of mitophagy is not sufficient to limit mitochondrial damage, leading to accumulation of dysfunctional mitochondria, producing increasing levels of ROS.

One of the most interesting phenomena that indirectly suggests a crucial role of oxidative stress in regulating mitophagy during aging is caloric restriction (CR). Caloric restriction is known to extend lifespan. Almost every study performed so far indicates that mtROS production is lower in liver, brain, heart, and other tissues of long-lived than of short-lived species [102], and that long-term CR reduces the rate of mtROS generation, and extends lifespan in different animal models, including *C. elegans* and mice. Not only CR prolongs lifespan in mice, but it also attenuates the effects of aging on different tissues, including skeletal muscle and myocardium [103]. In these animal models, CR causes the activation of AMPK-ULK1 pathway, which determines the removal of damaged mitochondria via mitophagy [97,98,99]. Aged mice kept for 20 weeks on a CR diet showed normal, not damaged mitochondria, low levels of oxidative stress and low levels of PINK1 in kidney, suggesting that CR can mitigate the mitochondrial damage observed with age, and making activation of the PINK1 pathway no longer necessary [104].

In some models, caloric restriction cannot be maintained for a long time. However, the use of molecules that mimics some of their effects can help in understanding the impact of CR on mitophagy [105]. An example of these molecules is spermidine, a dietary compound that has been shown to extend lifespan through induction of autophagy in *S. cerevisiae*, *C. elegans* and *D. melanogaster* [106]. Increased levels of Parkin-positive mitochondria have been found in aged hearts along with lower levels of Nrf1, a crucial factor for mitochondrial biogenesis, reduced Drp1-mediated mitochondrial fission, and formation of enlarged mitochondria [107]; spermidine feeding promotes protective autophagy and mitophagy in cardiomyocytes, counteracting mitochondrial respiration decline observed with aging [108]. Although it has not been shown that this effect on mitophagy is mediated by reduction or mtROS generation, the strict correlation between CR and mtROS suggests that this could be the case, at least in part. Notably, induction of autophagy by resveratrol, another molecule mimicking CR, is dependent on the nicotinamide adenine dinucleotide-dependent deacetylase sirtuin 1 (SIRT1) [109], a factor that is upregulated in response to oxidative stress in the heart [110]. Accordingly, moderate expression of Sirt1 induces resistance to oxidative stress and counteracts aging in mice [110]. Interestingly, in centenarians—exceptional humans who reach the age of 100 years or more—a decreased mitophagy has been observed, even in the presence of oxidative stress. This phenomenon is accompanied by a sort of “mitochondrial hypertrophy” within the cell that help to keep a full bioenergetic competence, even in the presence of OXPHOS defects [86].

## 5. Mitophagy and Oxidative Stress: Insights from Age-Related Diseases

The efficacy of the mitochondrial respiratory chain tends to diminish with aging, with a reduction in ATP synthesis and increase in the production of ROS [111]. Thus, cellular damage caused at least in part by chronic oxidative stress can accumulate, and autophagic and mitophagic pathways can become overwhelmed, particularly in non-dividing, high energy demanding cells such as neurons. As a consequence, cortical degeneration is commonly observed in aging. Thus, it is not surprising that many age-related diseases, such as AD or PD, show the simultaneous occurrence of chronic oxidative stress, mitochondrial dysfunctions and impaired mitophagy. The study of mitophagy in these pathologies, as well as in other diseases characterized by accelerated aging, provided crucial information about the mechanistic connections between mitophagy and oxidative stress during aging.

### 5.1. Mitophagy Defects and Oxidative Stress in Premature Aging Diseases

The observations concerning mitophagy defects in monogenic diseases characterized by premature aging are of particular interest to understand the interconnections between mitophagy and ROS. Loss of mitophagy was first described in Cockayne syndrome (CS), a progeroid syndrome characterized by progressive neurodegeneration that resembles that observed in mitochondrial disorders [112]. Mutations in CS complementation group B (CSB) gene cause the 80% of CS cases. It has been shown that CSB deficient cells are characterized by increased mitochondrial content, higher MMP and sustained production of ROS [113], accompanied by higher spare respiratory capacity and increased oxygen consumption rate. These changes did not appear to be related to increased mitochondrial biogenesis, but rather to an impairment of mitophagy that reduces the turnover of damaged mitochondria. This impairment is likely due to a reduced activity of PGC-1α, which is needed to the transcription of genes encoding uncoupling proteins (UCP). A lower UCP expression increases MMP and impairs PINK1-mediated mitophagy. In agreement with this hypothesis, the overexpression of UCP2 rescues mitophagic defects in CS [114]. A similar phenotype was reported in ATM deficient cells [115]: an increased oxygen consumption rate was associated with higher mitochondrial mass, higher ROS levels and decreased mitophagy. As ATM is present in mitochondria, it is possible that CSB and ATM contribute to a mtDNA damage response pathway by enhancing mitophagy. Notably, the same phenotype (higher mitochondrial metabolism, MMP and ROS formation, along with impaired mitophagy) has been found in related DNA repair disorder xeroderma pigmentosum group A [116].

### 5.2. Mitophagy and Oxidative Stress in Alzheimer’s Disease

AD represents a paradigmatic example of age-related, multifactorial neurodegenerative disease. AD occurs in two forms: a familial early-onset and a sporadic, late-onset form, and is the most common cause of dementia, accounting for 50–75 % of cases [117]. Early onset AD, which represents around 5% of AD total cases, is caused by highly penetrant mutations of few genes, *PSEN1*, *PSEN2*, and *APP* [118] whereas age-related factors are responsible for disease process and clinical symptoms. AD is characterized by progressive accumulation of extracellular aggregates of the amyloid-β peptide (Aβ), which are generated from cleavage of the membrane-bound amyloid precursor protein (APP), and aggregates of tau proteins, which form neurofibrillary tangles in the cytoplasm. Both soluble Aβ and abnormally phosphorylated tau can directly impair mitochondrial functions.

Multiple lines of evidence indicate that mitophagy is involved in neurodegeneration observed in AD. ROS are among the players that drive mitophagy impairment, and markers of oxidative stress (such as protein carbonylation, lipid oxidation, and the oxidation of the mtDNA) that are increased with age, appears to be particularly evident in AD [119]. Consistently, enhanced oxidative stress was observed in animal models of AD [120,121] and mtROS are sufficient to trigger Aβ production in vitro and in vivo [122]. Abnormal mitophagy in AD patient brains have been evidenced by autophagic accumulation of mitochondria in vulnerable AD neurons [110] and then confirmed by different groups [123]. The overexpression of mutant APP (mAPP) in mouse primary hippocampal cells results in higher expression of mitochondrial fission genes, DRP1, and FISs1 and decreased levels of fusion genes (MFN1, MFN2, and OPA1) as well as of autophagy (ATG5 and LC3BI, LC3BII) and mitophagy (PINK1, TERT, BCL2, and BNIP3L) genes at the mRNA and protein level [124], suggesting that the initiation and cargo recognition component of mitophagy is inhibited by Aβ.

The involvement of PINK1/Parkin-dependent mitophagy in AD pathogenesis have been intensively studied in the last years. Progressive Aβ accumulation and subsequent mitochondrial damage strongly induce PINK1/Parkin pathway in animal models of AD, and its upregulation has been observed in AD patient brains [125,126]. Furthermore, cytosolic Parkin is depleted in AD brains during disease progression, resulting in mitophagic impairment and augmented mitochondrial defects. In neurons bearing a mutant hAPP, an increased recruitment of cytosolic Parkin to depolarized mitochondria has been observed in the absence of MMP dissipation reagents [127]. Moreover, Parkin translocation has been observed mainly in the somatodendritic regions of the cells. This imbalanced recruitment leads to a decreased anterograde and increased retrograde mitochondrial axonal transport. Along with the observation that mitophagy is enhanced in AD brains, accompanied by depletion of cytosolic Parkin over disease progression, these data suggest that impaired mitophagy significantly contributes to the accumulation of dysfunctional mitochondria in AD-affected neurons [127]. In agreement with these observations, skin fibroblasts and brain biopsies from AD patients showed high levels of oxidized proteins, which suggests the presence of mitochondrial damage caused by oxidative stress, low Parkin levels and accumulation of PINK1 [128]. The overexpression of Parkin in cultured patients’ fibroblasts restored mitophagy, as evidenced by decreased PINK1 and accumulation of defective mitochondria, and recovery of MMP.

As far as the effects of abnormal Tau on mitophagy are concerned, first studies showed that the destabilization of microtubule networks and interruption of organelle migration determines accumulation of damaged organelles in the soma of neurons. In brains from AD patients with increased levels of Tau, an increase in the levels of different mitophagy markers has been observed, suggesting a mitophagy deficit within cells. The overexpression of hTau in a cellular model determines an increase of the MMP, associated with a decrease in the localization of Parkin to the mitochondria [129]. Tau has been shown to interact with Parkin and inhibit its translocation to defective mitochondria by sequestering it in the cytosol in neuroblastoma cells [130]. In a transgenic mouse model of AD, excessive levels of Tau can induce mitophagy by increasing MMP and Parkin levels [129]. Tau can also interact with DRP1, suggesting that it can contribute to increased mitochondrial fragmentation observed in AD [131]. In old transgenic tau mice bearing P301L mutation, increased levels of the fission proteins DRP1 and FIS1 and decreased levels of mitochondrial fusion proteins, MFNn1, MFN2, and OPA1 has been observed in the hippocampus. This change was associated with higher levels of mtROS and lipid peroxidation mice [132]. Although no data concerning mitophagy has been provided in this study, it is conceivable that the alterations of mitochondrial dynamics impact on mitophagy, and contribute to impaired capability to remove damaged mitochondria, which resulted more prone to produce ROS.

Finally, changes in the cardiolipin profile of synaptic mitochondria have been observed in a mouse model of AD, which were associated with mitochondrial dysfunction, in a way similar to what observed during aging in rat hearts [35,133,134]. Even if direct evidence has not been provided, this observation could also suggest that cardiolipin-related mitophagy could also be impaired in AD [135].

Defects in the proteolytic activities of lysosomes can impair mitophagy. Lysosomal defects have been repeatedly observed in brains specimens from AD patients. Studies in mouse models highlighted the importance of lysosome functionality in AD pathogenesis, as suppression of lysosomal proteolysis mimics AD neuropathology, while restoring normal lysosomal proteolysis and autophagy efficiency in AD mouse models improves neuronal function and cognitive performance, [136,137]. Accordingly, mutations of PSEN1—one of the genes causing early onset AD—in combination with ApoE4, a key genetic risk factor of AD, disrupt lysosomal function [138]. Other factors, including Aβ peptides, ROS, and oxidized lipids and lipoproteins, can also impair lysosomal proteolysis. Lysosomal deficits in AD have been also attributed to defects in protease targeting to lysosomes [139]. As a whole, these lysosomal defects reduce proteolytic removal of defective mitochondria, along with other autophagic cargoes, in neurons of AD patients [139]. Therefore, increased Parkin association with mitochondria and abnormal mitochondrial retention within lysosomes observed in AD neurons of patients, as well as in cells overexpressing mutant APP, could also be due to lysosomal deficiency [139].

Overall, these studies demonstrate that mitophagy impairment is clearly involved in AD pathogenesis, and contributes to the progressive loss of mitochondrial functionality observed in AD progression. To what extent the functional alteration of mitochondria associated with age is crucial for the development of AD, or to what extent it represents a concurrent but secondary phenomenon is still under discussion. According to the so-called “mitochondrial cascade hypothesis”, mitochondria could represent the primary generator of AD [140]: since mitochondrial function declines during aging, it connects AD and aging by explaining why advanced age is the greatest AD risk factor. In physiological aging, an equilibrium exists between mtROS, mitochondrial damage and removal of dysfunctional mitochondria via mitophagy. In AD progression, the decrease below a critical threshold of mitochondrial function associated with age starts the events leading to the accumulation of Aβ. Mitophagy impairment can contribute to overcome this threshold, and in turn impaired mitochondrial function and associated bioenergetic changes alter Aβ homeostasis and lead to an accumulation of Aβ.

### 5.3. Mitophagy and Oxidative Stress in Parkinson’s Disease

PD is the second most common progressive disorder of the CNS, that affects predominantly the dopaminergic neurons od the *substantia nigra* (SN) [141]. A typical sign of PD is the presence of the Lewy bodies, eosinophilic cytoplasmic inclusions in the SN [142]. PD is mainly sporadic and associated with aging, even if 5–15% is hereditary with an autosomal transmission, always with early onset [143]. In a way similar to AD, the early-onset form of PD is caused by gene mutations, whereas aging is the single, most important risk factor for the sporadic form. Indeed, the prevalence of PD is at 5% in people aged 80 years, at 2% in aged 65 years, and rare in aged 50 years or less [144,145]. The mechanisms at the basis of neuronal degeneration in PD have not been fully elucidated, but several lines of evidence suggest that deficiencies in mitochondrial homeostasis play a crucial role in neuronal degeneration characterizing PD pathogenesis [146]. The high request of energy of these cells is probably at the basis of their susceptibility to mitochondrial dysfunction [141] and both mitophagy impairment and oxidative stress have been proved to be involved in this process.

A possible involvement of mitophagy in the pathogenesis of PD was first suggested by a study showing that mitochondria accumulated abnormally in autophagosomes in neurons of patients with PD and Lewy Body Dementia (LBD) [147]. Numerous studies have described defects in mitophagy and an overall mitochondrial impairment with consequent increased ROS production in neurons of PD patients and/or in models [146,148,149,150,151,152,153,154,155,156]. Although oxidative stress is described as a key regulator of the neurodegenerative process in all forms of PD [146,157], it is not clear yet if the increase of ROS is a causative factor or a consequence of cells degeneration. Nevertheless, investigation in early-stage PD patients showed that oxidative stress is arising during the initial stages of the disease before the neuron loss, supporting the idea that ROS could be the cause of neuronal degeneration [158]. As oxidative stress is observed during physiological aging, molecular alterations occurring during PD could aggravate the imbalance between ROS production and scavenging observed with age, leading to nigral neurodegeneration [145].

The observations made on early onset, recessive familial PD are of particular interest to understand how impairment of mitophagy affects the delicate balance between damage caused by oxidative stress and mitochondrial turnover maintaining the organelle homeostasis. Early onset, recessive familial PD can be caused by mutations in the genes *Park2* (*Parkin*), *Park6* (*Pink1*), or *Park7* (*DJ-1*), among others [125,159,160,161]. All three proteins are crucial in resistance to oxidative stress and to maintain mitochondrial functions [125]. While the role of PINK1 and Parkin in mitophagy is clearly established, the possible involvement and the precise function of DJ-1 in this process is still a matter of discussion. DJ-1 is involved in anti-oxidative, anti-inflammatory, and anti-apoptotic pathways, and can protect *substantia nigra* from oxidative stress during PD onset. Missense mutations in this gene cause a very rare autosomal recessive PD, often with early onset [162], and fibroblasts and lymphoblasts from PD patients with mutated DJ-1 showed fragmentated mitochondria, revealing a role of DJ-1 in mitochondrial dynamics [163,164,165]. Interestingly, overexpression of PINK1 and Parkin rescues the aberrant mitochondrial phenotype observed in DJ-1 deficient cells, suggesting that DJ-1 functions in mitophagy are partially redundant [164].

Alpha-Synuclein, encoded by the *SNCA* gene, plays a role in compartmentalization of neurotransmitters and synaptic vesicle recycling and it is mainly located in neurons, in presynaptic terminals [146,166,167,168,169,170]. Multiplications and mutations in *SNCA* gene are related to autosomal dominant PD. Alpha-Synuclein is a natively unfolded protein, without a stable structure in aqueous solutions, and its aggregation represents a hallmark of PD [146,171]. Indeed, alpha-Synuclein fibrils are the main component of Lewy bodies. Mutated or high amount of protein can constitute aggregations and amyloid fibrils [172,173,174,175]. Numerous in vivo and in vitro studies connect oxidative stress with the formation of α-Synuclein aggregations and fibrils, supporting the idea that an unbalance redox state in brain may contribute to neurodegeneration [176,177]. Neurotoxins such as rotenone or MPP^+^ (metabolite of 1-methyl-4-phenyl-1,2,3,6-tetrahydropyridine, MPTP) capable of inhibit mitochondrial complex I producing ROS have been shown to increase α-Synuclein in vitro and in vivo; a study revealed that this effect is due to the derepression of microRNAs (miRNAs) capable of inhibit α-Synuclein mRNA expression, leading to a *de novo* translation [172]. Moreover, an increase of α-Synuclein aggregation has been observed in a transgenic mouse overexpressing α-Synuclein in the presence of oxidative stress due to haploinsufficiency of SOD2 [178]. Furthermore, α-Synuclein misfolding is responsible of increasing in ROS production, triggering a vicious cycle that in turn leads to neurodegeneration [179,180].

LRKK2 is a kinase enzyme codified by the LRRK2 gene and mutations in LRRK2 are responsible for 1–2% of total PD cases and about 5% of total familial cases, even if LRKK2 represent also a risk locus for sporadic PD [181,182,183]. It has been showed that LRKK2 can interact with Miro, a protein of the OMM responsible for the link between microtubules and the mitochondrial surface and involved in mitochondria mobility [184,185,186]. Interaction of LRKK2 with Miro targets it for degradation and triggers mitophagy. In a study performed on fibroblasts from sporadic and familial LRRK2-mutated PD patients treated with CCCP (a mitochondrial uncoupler), Miro degradation, and the subsequent mitochondrial clearance were compromised. Delaying and impairment of mitochondrial clearance were also observed in induced pluripotent stem cell (iPSC)-derived neurons from PD patients with LRKK2G2019S mutations treated with antimycin A, an inhibitor of complex III able to start mitophagy. This demonstrated that mutations in LRRK2 could delay Miro degradation and the onset of mitophagy, leading to the increase of ROS, followed by cell death [187]. In another study, the effects of mutated PINK1 have been analyzed in dopaminergic neurons derived from iPS cells from skin fibroblasts of PD patients. Although iPSC were treated with valinomycin, a potassium ionophore capable of dissipate the transmembrane electrochemical gradients, they showed impaired Parkin translocation to mitochondria with an increase of mitochondrial copy number [188].

Multiple lines of evidence suggest that the imbalance of acetylases and deacetylases activity also impacts mitophagy regulation in PD pathogenesis, either in idiopathic or familiar forms of the disease. Hyperacetylation of SOD1 has been observed in post-mortem midbrains from PD patients [189]. SIRT3 overexpression, or administration of Nicotinamide Riboside (NR), a NAD+ precursor, counteracts the degeneration of dopaminergic neurons in PD [190]. CR, which is known to induce SIRT3, reduces neurodegeneration in animal models of both PD and AD [191]. Decreased sirtuin deacetylase activity was observed in iPSC-derived dopaminergic neurons from patients bearing the G2019S LRRK2 mutation [192]. Fibroblasts from PD patients with the same mutation displays increased mitophagy, due to the activation of SIRT3, clearly suggesting that impaired SIRT-induced mitophagy plays a major role in the pathogenesis of this form of early-onset PD. Conversely, idiopathic PD exhibits a reduced capability to remove defective mitochondria, associated with higher levels or ROS production reactive oxygen species (ROS) [71].

**Figure 2:**
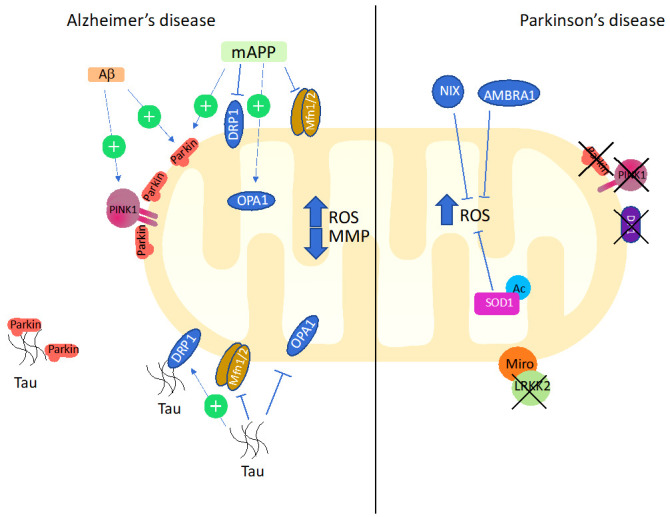
Main alterations of factors modulating mitophagy observed in AD and PD. In AD, Aβ accumulation upregulated PINK/Parkin pathway. Mutant human APP increases recruitment of Parkin to depolarized mitochondria. mAPP also causes upregulation of mitochondrial fission genes DRP1, and decrease of fusion genes MFN1/2 and OPA1. Tau interacts with Parkin and inhibits its translocation to defective mitochondria. Furthermore, it upregulates fission proteins and inhibits fusion proteins. In PD, defective mitophagy is determined by loss of function of Parkin, PINK1, and DJ- 1; their dysfunction is associated with high levels of ROS. Loss of LRKK2 blocks the degradation of the outer membrane protein Miro and triggers mitophagy. In this context, NIX and AMBRA, can limit the excessive production of ROS. See text for details.

The crucial role of oxidative stress and mitophagy in the pathogenesis of PD has been also proved by the observation that NIX and AMBRA1 can help in delaying cell death in PD by exerting an antioxidant action. When PINK1-mediated mitophagy is abrogated in PD dopaminergic neurons, NIX can stimulate the removal of damaged mitochondria, so preserving dopaminergic neurons. Phorbol 12-myristate 13-acetate (PMA) induces NIX expression, and leads to ROS production in the same cells, clearly suggesting that induction of mitophagy helps in reducing oxidative stress [193]. In a similar model in which PINK1-mediated mitophagy is not functional, because of PINK1 or PARK2 mutations, AMBRA1 can rescue mitophagy [194] and reduce cell death in vitro induced by rotenone or 6-OHDA treatments, by limiting selectively the source of oxidative stress [195]. The main alterations of factors modulating mitophagy observed in AD and PD are depicted in Figure 2, while Table 1 summarizes the main findings described above concerning changes of mitophagy observed during aging or in PD and AD, which are related to oxidative stress.

## 6. Conclusions

**Figure 3:**
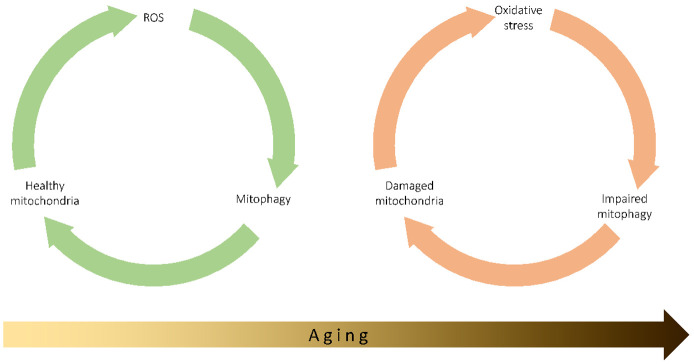
In normal conditions, ROS physiologically produced in the cell, and particularly by mitochondria, can induce mitophagy, which contributes to the normal homeostasis of the cells by removing damaged mitochondria, so maintaining the organelles healthy. The progressive increase in ROS production observed with age can lead to chronic oxidative stress, which in turn impairs mitophagy and reduces the capability to remove damaged mitochondria. Damaged organelles further produced ROS, so keeping a vicious cycle active.

The interplay between mitophagy, ROS production, and aging is complex and far from being completely elucidated. The central role of ROS production and consequent damage to mitochondria in the aging process has been clearly established in the last 50 years, despite some objections to this theory over the past 15 years [212], and mitophagy is a key mechanism for mitochondrial quality and quantity control, as it limits the production of ROS, the damage to mtDNA of transmembrane potential loss and the decrease in ATP production. The data and observations discussed in this review indicate that the imbalance of the delicate equilibrium among mitophagy, ROS production, and mitochondrial damage can start, drive, or accelerate the aging process, either in physiological or pathological conditions (Figure 3). It remains to be determined which is the prime mover of this imbalance, i.e., whether it is the mitochondrial damage caused by ROS that initiates the dysregulation of mitophagy, thus activating a vicious circle that leads to the reduced ability to remove damaged mitochondria, and further damage from ROS, or if, on the other hand, an alteration in the regulation of mitophagy constitutes one of the initial events leading to the main of the excessive production of ROS.

## Figures and Tables

**Table 1:** Changes of mitophagy observed with age or in PD and AD, which are related to oxidative stress.

**Mitophagy Pathway or Protein**	**Age Related Changes**	**Changes in PD or AD**
**Pink/Parkin**	Depletion of Pink1 and Parkin leads to hallmarks of senescence in ISCs (Intestinal stem cells) of *D. melanogaster,* including high ROS levels [94]; Overexpression of Pink1 or Parkin extends lifespan of *D. melanogaster* [95]; Parkin overexpression attenuates molecular and biochemical markers of aging, extending lifespan in *D. melanogaster* [196] mRNA levels of BNIP3, PINK1, Parkin and NIX, and the protein levels of BNIP3, PINK1 and Parkin decrease in the mouse auditory cortex with aging [197]; Parkin levels are diminished in atrophied muscles of elderly men [98]; Parkin overexpression attenuates the effects of advanced aging on myocardial function in transgenic mice [198] Parkin overexpression attenuates aging-dependent loss of muscle strength and mass in transgenic mice [97].	Upregulation of the PINK1/parkin pathway were showed in AD patients’ brain [119,120]; Depletion of Parkin during AD progression were found in AD patients’ brain [121]; Low levels of the Parkin protein were identified in skin fibroblasts and brain biopsies from AD patients [122]; Increased levels of Parkin were revealed in a transgenic mouse model [123]; Homozygous or compound heterozygous mutations and the consequent loss of function of Parkin and PINK1 genes are the main cause of recessive early-onset PD [159,119]; Mutations in PINK1 gene are also a rare source of sporadic early-onset PD [160].
**Cardiolipin**	Cardiolipin levels in mitochondria decrease with aging [134]; Changes to cardiolipin content and oxidative damage have been related to aging in hearts of rats; no direct evidence of cardiolipin-mitophagy impairment has been provided [35,133].	Changes in the cardiolipin profile were described in mouse model of AD [126]; Correlation between oxidative damage of CL by ROS and pathogenesis of PD, likely because of the impairment of mitophagy caused by damaged CL. No direct evidence is provided [199].
**DJ-1**	DJ-1 mutants in *D. melanogaster* exhibit lifespan shortening and sensitivity to oxidative stress; Repressed during aging in rat thymus tissues [200].	Immunostaining revealed high levels of DJ-1 protein in hippocampal pyramidal neurons and astrocytes of AD brains [201]; Mutations in DJ-1 gene are cause of autosomal recessive PD [161]; Fibroblasts and lymphoblasts from PD patients with mutated DJ-1 showed fragmented mitochondria [151,153]. Mutations in Dj-1 impaired protection against oxidative stress, a key regulator of the neurodegenerative process in PD and AD [202,203,204,205,206,207,208,209,210]
**BNIP3**	mRNA levels of BNIP3, PINK1, Parkin and NIX, and the protein levels of BNIP3, PINK1 and Parkin in the mouse auditory cortex decrease with aging [197].	No data available
**MFN1**	Age-related increase of MFN1 and OPA1 in cultured fibroblasts.	Increased expression in neurons from patients with AD.
**MFN1/MFN2**	MFN2 expression is higher in rat and human chondrocytes during aging and OA (osteoarthritis) [101] MFN2 decreases during aging in mouse skeletal muscle [99].	Reduced levels of MFN1, MFN2 and OPA1 were found in aged tau mice [132]; Changes of MFN1 and MFN2 were identified both in the PrP-hAPP/hPS1 AD mouse model brains and in an SH-SY5Y cell model of early-onset AD [211]. Decreased levels of mitochondrial fusion proteins, MFN1, MFN2 and OPA1 were found in 12-month-old tau mice relative to age-matched WT mice [132].

## Abbreviations

AD	Alzheimer’s disease
AMBRA1	Autophagy and beclin 1 regulator 1
APP	Amyloid precursor protein
ARIH1	Ariadne RBR E3 ubiquitin ligase homolog 1
ATG	Autophagy-related protein
ATM	Ataxia-telangiectasia mutated
Aβ	Amyloid-β peptide
BCL2L13	BCL2 like 13
BNIP3	BCL2 interacting protein 3
CI	Complex I
CIII	Complex III
CK2	Casein kinase II
CL	Cardiolipin
CR	Caloric restriction
CS	Cockayne syndrome
CSB	CS complementation group B
CuZnSOD (SOD1)	Copper-zinc SOD
DNM1L	Dynamin-1-like protein
Drp1	Dynamin-1-like protein
FKBP8	FKBP prolyl isomerase 8
FUNDC1	FUN14 domain containing 1
Gp78	Glycoprotein 78
GPx1	Glutathione peroxidase 1
Grx2	Glutaredoxin 2
GSNOR	S-nitrosoglutathione reductase
HAT	Histone acetyltransferase
HDAC	Histone deacetylases
HIF1a	Hypoxia-inducible factor 1-alpha
HUWE1	HECT, UBA and WWE domain-containing protein 1
IMM	Mitochondrial inner membrane
iPSC	induced pluripotent stem cell
IR	Ischemia/reperfusion
Keap1	Kelch-like ECH-associated protein 1
LBD	Lewy Body Dementia
LIR	LC3 interacting region
mAPP	Mutant APP
MnSOD (SOD2)	Manganese superoxide dismutase
MPP	Matrix processing peptidase
MsrB2	Methionine sulfoxide reductase B2
mtKR	Mt KillerRed
MMP	Mitochondrial membrane potential
MTS	Mitochondrial targeting sequence
MUL1	Mitochondrial E3 ubiquitin protein ligase 1
NAD	Nicotinamide adenine dinucleotide
NBR1	Neighbor of BRCA1 gene 1
NDP52	Nuclear domain 10 protein 52
NIX (BNIP3-like, NIX/BNIP3L)	NIP3-like protein X
NK	Natural killer
NR	Nicotinamide riboflavide
Nrf2	Nuclear factor (erythroid-derived 2)-like 2 transcription factor
OMM	Mitochondrial outer membrane
OPA1	Dynamin-like 120 kDa protein
OPTN	Optineurin
p62/SQSTM1	Sequestosome-1
PARL	Presenilin-associated rhomboid like
PD	Parkinson’s disease
PGAM5	Phosphoglycerate mutase 5
PHD	Prolyl hydroxylase
PINK1	Phosphatase and tensin homologue (PTEN)-induced putative kinase 1
PMA	Phorbol 12-myristate 13-acetate
PRDX6	Peroxiredoxin-6
Prx3	Peroxiredoxin-3
Prx5	Peroxiredoxin-5
Rbx1	E3 ubiquitin-protein ligase RBX1
ROS	Reactive oxygen species
SENPs	Sentrin/SUMO specific proteases
SIAH1	E3 ubiquitin-protein ligase SIAH1
SIRT1	Nicotinamide adenine dinucleotide–dependent deacetylase sirtuin 1
SMURF1	SMAD ubiquitination regulatory factor 1
SNc	Substantia nigra pars compacta
SNCA	Alpha-Synuclein
TAX1BP1	TAX1 binding protein 1
TIM	Translocase of the inner membrane
TOM	Translocase of the outer membrane
UCP	Uncoupling proteins
ULK1	Unc-51-like autophagy activating kinase 1
